# Investigation of Cutting Forces and Temperature in Face Milling of Wood–Plastic Composite Using Radial Basis Function Neural Network

**DOI:** 10.3390/ma18204731

**Published:** 2025-10-15

**Authors:** Feng Ji, Zhaolong Zhu

**Affiliations:** 1 Co-Innovation Center of Efficient Processing and Utilization of Forest Resources, Nanjing Forestry University, Nanjing 210037, China; njfuzzlong@outlook.com; 2 Physical Education Department, Nanjing Forestry University, Nanjing 210037, China; 3 College of Furnishings and Industrial Design, Nanjing Forestry University, Nanjing 210037, China

**Keywords:** wood plastic composite, face milling, radial basis function neural network, cutting force, cutting temperature, predictive modeling

## Abstract

Wood–plastic composite (WPC) is being increasingly adopted in construction and furniture applications due to its durability and recyclability. This study investigates face-milling responses—resultant cutting force and cutting temperature—under systematically varied cutting parameters, and develops a radial basis function neural network for predictive modeling. Experiments were conducted on a computer numerical control machining center using a polycrystalline diamond end-milling cutter for face milling with fixed axial depth of cut. Feed speed, radial depth of cut, and spindle speed were selected as input factors. The results indicate that feed speed and radial depth of cut generally increase all force components, whereas higher spindle speed tends to reduce force magnitudes while elevating temperature. The radial basis function neural network yields acceptable accuracy for resultant cutting force (coefficient of determination R^2^ ≈ 0.91) and acceptable accuracy for cutting temperature (R^2^ ≈ 0.81). These findings demonstrate the feasibility of radial basis function neural network based prediction for WPC face milling and provide guidance for parameter selection.

## 1. Introduction

With rapid advanced material modification technology, wood–plastic composite (WPC) serves as an environmentally friendly and sustainable new material widely used in areas such as construction, furniture, and automotive [1]. The characteristics of WPC endow it with the advantages of natural wood and plastic, including corrosion resistance, ease of processing, and moisture resistance, making it a substitute for traditional building materials in many application scenarios [2]. However, despite the great progress WPC has made in various fields, its manufacturing process still faces a series of technical challenges, including issues related to production efficiency, raw material utilization, and product quality [3,4].

Currently, research on the cutting performance of WPC is predominantly focused on peripheral milling, mainly including cutting force, temperature, and surface roughness. Zhu et al. [5] explored the frictional behavior of WPC; their work showed friction is the main factor affecting cutting force, temperature, and surface roughness. The cutting force, temperature, and surface roughness of WPC during diamond peripheral milling was explored by Wang et al. [6] and Guo et al. [7], who found that increased spindle speed and rake angle can improve cutting performance in terms of lower cutting force, temperature, and surface roughness. Meanwhile, built-up was examined by Zhu et al. [8]; they found that an increase of cutting force and temperature by high cutting speed and depth led to the built-up edge, the generation of which changed the work angle, thereby resulting in a poorer machined surface with high roughness. In general, cutting force, heat, and quality are crucial indicators influencing the cutting performance of WPC. Scientific cutting parameters play a key role in reducing cutting force and heat while improving surface quality [9].

Face milling, a specific milling technique, focuses on machining the surface of a workpiece perpendicular to the cutter axis. This process is commonly employed for flat surfaces, creating a smooth finish and precise geometric features. Face milling has proven that it has great cutting performance [10,11,12]. Firstly, it enables efficient material removal, enhancing productivity in manufacturing. Secondly, face milling produces superior surface finishes, meeting stringent quality requirements in industries such as aerospace and automotive. Additionally, the process allows for the machining of large workpieces with high precision, ensuring dimensional accuracy [13,14,15]. Recently, face milling found extensive application across various industries. In automotive manufacturing, it is utilized to produce engine blocks, transmission cases, and other components requiring flat and precise surfaces. In the aerospace sector, face milling is crucial for crafting aircraft components with tight tolerances. The versatility of face milling extends to general machining tasks, including the production of molds and dies in the tool and die industry [16,17].

Beyond physics-based models, artificial neural networks (ANNs) and other machine-learning (ML) methods have been increasingly used to predict machining responses and to support process optimization in materials manufacturing. The previous literature has demonstrated ML-based prediction of mechanical, thermal, and tribological properties across a range of materials systems. To situate the present work, the introduction includes a concise overview of ANN applications for materials and machining response prediction, followed by the study objectives.

Prior ANN studies have been used to predict machining responses across metals and composites; however, data regimes, feature choices, and validation practice vary widely. Many studies report single-response models and limited calibration reporting, complicating reproducibility. Here, this work focuses on dual responses (force and temperature) under small-sample constraints, providing a transparent training protocol and reporting R^2^ as the primary metric, which facilitates comparison and reuse.

This work addresses a clear gap in the literature: while peripheral milling of WPC has been widely studied, face milling of WPC remains underexplored despite its industrial relevance for flatness and productivity. This study (i) quantifies how feed speed, radial depth of cut, and spindle speed affect force components and cutting temperature in WPC face milling, (ii) develops a lightweight yet robust RBF-NN protocol tailored to small datasets with explicit regularization and early stopping, and (iii) reports reproducible details and practical ranges that balance force reduction and thermal load.

## 2. Materials and Method

### 2.1. Materials

The WPC material was supplied by Guofeng Wood–Plastic Composite Co., Ltd. (Hefei, China), composed of poplar powder and recycled polyvinyl chloride (PVC) in a mass ratio of 3:2. Additives included 2% lubricant (ethylene bis-streamside) and 1% pigment (titanium dioxide). The physical–mechanical properties in Table 1 were provided by the supplier. The dimension of the workpiece is 120 mm × 80 mm × 10 mm.

The cutting tool was a polycrystalline diamond (PCD) face-milling cutter with eight teeth, diameter D (10 mm), rake angle γ (10°), clearance angle α (12°). The cutting-edge material properties are listed in Table 2. The PCD face-milling tool was uncoated and supplied by Leitz Tooling System Co., Ltd (Nanjing, China). New edges were used at the start; after each set of runs, the tool was inspected, and milling continued only if flank wear VB ≤ 0.2 mm, measured by an optical microscope (SZX16, Olympus, Co., Ltd., Tokyo, Japan). The average edge radius measured about 10 μm.

### 2.2. Experimental Design and Parameters

**Figure 1:**
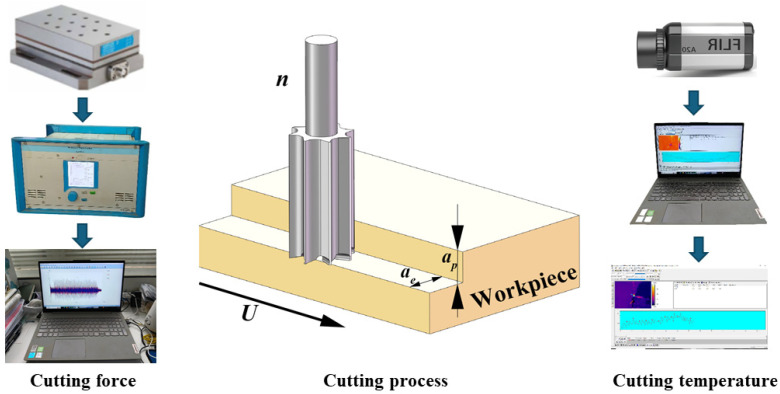
Cutting experiment.

Face-milling experiments were performed on a CNC machining center (MGK01, Nanxing Machinery Co., Ltd., Dongguan, China). The axial depth of cut was fixed at *a_p_* = 5 mm (Figure 1). The factors investigated were feed speed *U* (m/min), radial depth of cut *a_e_* (mm), and spindle speed *n* (rpm). The combinations are provided in Table 3. The ranges reflect preliminary stability trials within the machine’s window and values reported for WPC in the literature [4,5,7]; they also correspond to settings used in the industrial context. Specimen clamping ensured rigid support on a quartz-type dynamometer. Between runs, the tool was inspected to confirm no abnormal damage had been done.

### 2.3. Force and Temperature Measurement

The workpieces were fixed on a dynamometer (Kistler 9257B, Kistler Group, Winterthur, Switzerland) to collect the cutting force signal. Then, the signals were transmitted through a charge amplifier (Kistler 5070A, Kistler Group, Winterthur, Switzerland) to obtain three-direction cutting force, *F_x_* (normal force), *F_y_* (tangential force), and *F_z_* (lateral force). The resultant force (*F_R_*) was computed as follows [8]:

(1) $$F_{R}=\sqrt{F_{x}{\,}^{2}+F_{y}{\,}^{2}+F_{z}{\,}^{2}}$$

Cutting temperature was shot by an infrared imager (Thermo Vision A20-M, FLIR Systems Inc., Wilsonville, OR, USA) with the cutting process; then, the surface roughness of the machined workpieces was measured by a surface roughness meter (S-NEX001sd, Tokyo Seimitsu Co., Ltd., Tokyo, Japan). Infrared temperature was recorded with emissivity set to 0.94. Emissivity was determined by matching the IR readings over a black electrical tape patch (ε ≈ 0.95) to the adjacent bare WPC area during a controlled heating–cooling cycle; the reflected apparent temperature was set from ambient using a crumpled-foil check. A calibrated spot IR thermometer served as a secondary non-contact reference.

Force signals were filtered to remove electrical noise (e.g., zero-phase Butterworth, cutoff 100–200 Hz depending on sampling frequency). Steady-state time windows were selected to compute average force components per run. Thermal data were averaged over a fixed area near the contact zone to obtain a representative cutting temperature.

### 2.4. RBF-NN Model and Training Protocol

This study uses a compact radial basis function neural network (RBF-NN) to map the cutting parameters x = [*U*, *a_e_*, *n*] to the responses y ∈ {Fx, Fy, Fz, FR, T}. The dataset comprised 27 runs, partitioned into training/validation/test as 21/3/3. Features were standardized to zero mean; unit variance was based on the training set. The training process was stopped when the mean squared error fell below 1 × 10^−5^ or after 1000 epochs.

Centers are initialized by k-means on standardized inputs. Each center is assigned a width based on local inter-center distances scaled by a factor. The output layer is estimated with ridge-regularized linear solving to improve stability and reduce variance. Hyper-parameters include number of centers (K), scaling (β), local width estimation with neighbor count (d), and ridge regularization (λ). A set (K, β, d, λ) was used to denote the hyper-parameters combination. For validation, their range was selected as K ∈ {6, 8, 10, 12}, β ∈ {0.8, 1.0, 1.2, 1.5}, d ∈ {3, 4, 5}, λ ∈ {1e-6, 1e-5, 1e-4, 1e-3, 1e-2} [18,19,20,21,22,23,24,25,26,27,28,29]. Because units and ranges differ across responses, predictive association was evaluated using the coefficient of determination (R^2^). Experimental summaries are plotted with 95% confidence intervals (*N* = 3 repeats per condition).

## 3. Results and Discussion

### 3.1. The Effect of Cutting Parameters on Cutting Force

**Figure 2:**
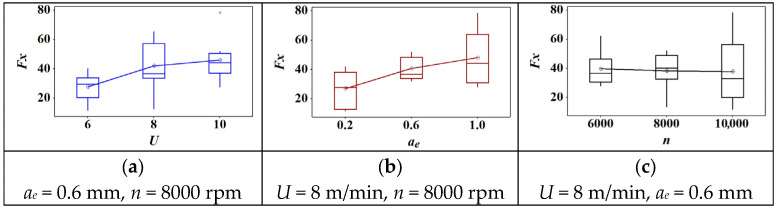
The effect of cutting parameters on normal force: (**a**) feed speed; (**b**) radial depth of cut; (**c**) spindle speed.

The box plot of cutting parameters versus normal force is shown in Figure 2. Firstly, there were no outliers in each graph. Second, the mean value of normal force increased with the increase of feed speed and cutting depth. However, the normal force presented a slightly decreased trend when the rotation speed increased. This indicated that feed speed and cutting depth have a positive impact on normal force. Notably, it could be difficult to decrease the normal force by increasing the rotation speed due to a slight reduction of force.

**Figure 3:**
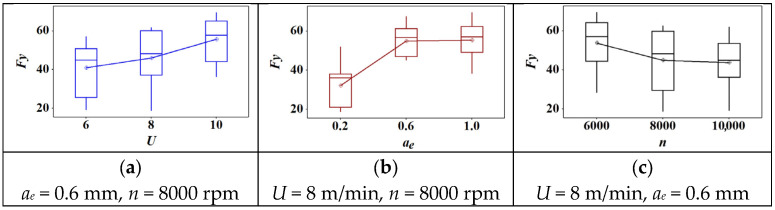
The effect of cutting parameters on the tangential force: (**a**) feed speed; (**b**) radial depth of cut; (**c**) spindle speed.

The box plot of cutting parameters versus tangential force is shown in Figure 3. Firstly, there were no outliers in each graph. Second, the mean value of tangential force increased with the increase of feed speed and cutting depth. However, the tangential force presented a decreasing trend with the increase of rotation speed. Furthermore, the increasing trend of tangential force was stable under the condition of increasing feed speed in Figure 3b. However, the tangential force showed a sharp increase when the cutting depth increased from 0.2 mm to 0.6 mm. Then, it showed an almost horizontal trend when the cutting depth continued to increase. In contrast, the tangential force presented a sharp decreasing trend when the rotation speed increased from 6000 r/min to 8000 r/min (Figure 3c). Then, it showed a slightly decreased trend when the rotation speed continued increasing to 10,000 r/min. Overall, a shallow cutting depth (0.2 mm) and a higher rotation speed (8000 r/min) can effectively decrease the tangential force.

**Figure 4:**
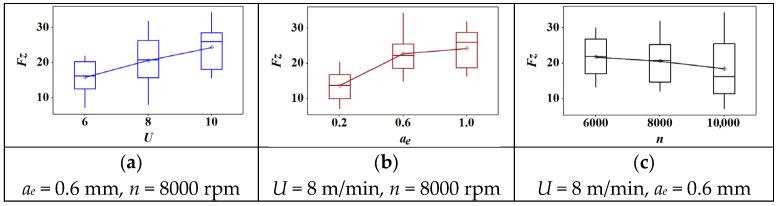
The effect of cutting parameters on the lateral force: (**a**) feed speed; (**b**) radial depth of cut; (**c**) spindle speed.

The box plot of cutting parameters versus lateral force is shown in Figure 4. Firstly, there were no outliers in each graph. Second, the mean value of lateral force increased with the increase of feed speed and cutting depth. Similarly, the lateral force showed a decreasing trend when the rotation speed increased. As shown in Figure 4a, the lateral force showed a near-linear growth with the increase of feed speed. However, it first showed a sharp increase when the cutting depth increased from 0.2 mm to 0.6 mm. Then, it showed a slight increase when the cutting depth reached 1.0 mm. When the rotation speed increased from 6000 r/min to 10,000 r/min, the lateral force presented a slightly decreasing trend. In general, cutting depth has the greatest effect on lateral force among these parameters. In other words, only a shallow cutting depth can effectively decrease the lateral force.

Across the tested range, both *U* and *a_e_* increased all force components, consistent with larger uncut chip thickness and material engagement. In contrast, higher *n* reduced force magnitudes, likely due to temperature-assisted softening of the PVC matrix and reduced average contact time per tooth. Among the tested factors, *a_e_* exhibited the strongest effect on lateral and tangential components, indicating sensitivity to chip width. The resultant force *F_R_* followed the same monotonic trends.

In WPC, the PVC-rich matrix exhibits viscoelastic softening with temperature, lowering effective deformation resistance as *n* rises; however, increases in *U* and *a_e_* raise the instantaneous uncut chip thickness and the contact length, thereby increasing the cutting load. The observed dominance of *a_e_* is therefore attributed to geometric engagement, whereas the mitigating role of *n* is attributed to a combination of shorter contact time and reduced frictional shear strength at elevated interface temperature.

### 3.2. The Effect of Cutting Parameters on Cutting Temperature

**Figure 5:**
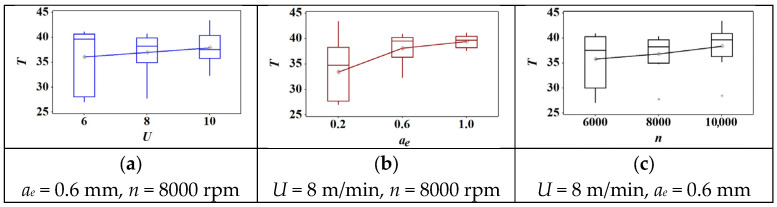
The effect of cutting parameters on the cutting temperature: (**a**) feed speed; (**b**) radial depth of cut; (**c**) spindle speed.

The box plot of cutting parameters versus cutting temperature is shown in Figure 5. Firstly, there were no outliers in each graph. Second, the mean value of cutting temperature increased with the increase of feed speed, cutting depth, and rotation speed. Furthermore, the increasing trend of cutting temperature presented a slight increase in feed speed and rotation speed. However, it first sharply increased when the cutting depth increased from 0.2 mm to 0.6 mm. Then, it showed a slight increase when the cutting depth reached 1.0 mm.

Generally, cutting temperature *T* increases with *U* and *a_e_* and also increases with *n* over the tested range. The growth with *n* occurs despite the reduction in forces, which indicates that frictional heating at a higher sliding velocity dominates the temperature field near the tool–chip interface.

Conditions that minimize force do not coincide with those minimizing *T*. Increasing *n* reduces forces but elevates *T*; increasing *a_e_* raises both force and *T*. Consequently, parameter selection must consider a force–temperature trade-off rather than a single optimum. For applications where thermal load is critical, combinations with moderate *U*, lower *a_e_*, and intermediate *n* provide a balanced regime.

### 3.3. RBF-NN Model for the Cutting Process

**Figure 6:**
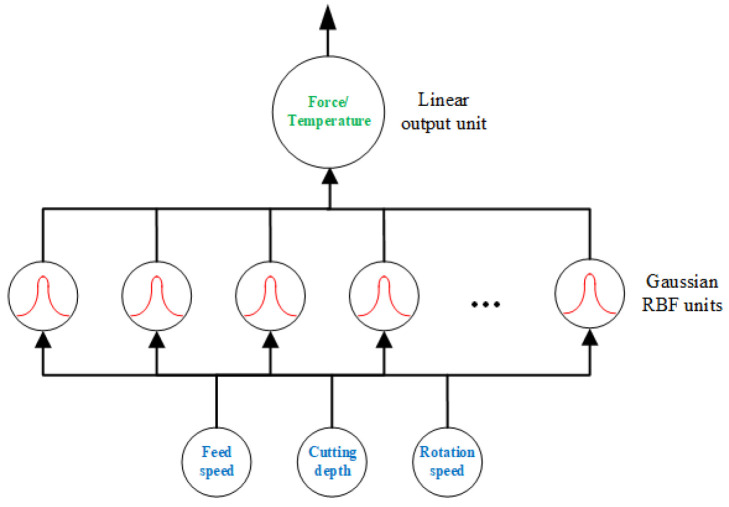
The architecture of the RBF model used in this work.

The framework of the RBF model for cutting force and temperature is shown in Figure 6. The input parameters were set as feed speed, cutting depth, and rotation speed. The output parameters were set as resultant cutting force and cutting temperature separately [30,31,32]. There were 27 groups which contained 21 groups for training data (approximately 80%), three groups for validation data (approximately 10%), and three groups for test data.

#### 3.3.1. RBF-NN Model for the Cutting Force

**Figure 7:**
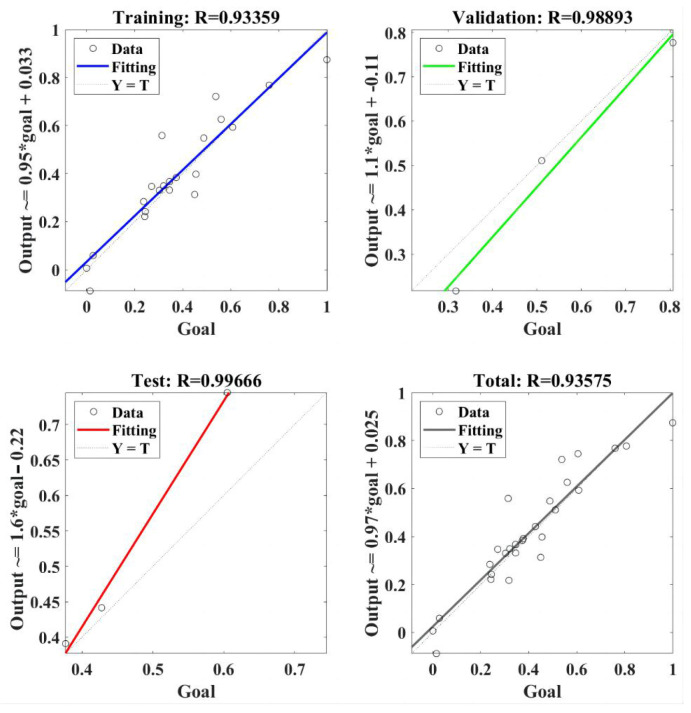
The regression of the RBF-NN model for the normal force.

The regression model of RBF-NN for the normal force is presented in Figure 7. The RBF-NN model was trained using selected data, and the value of correlation coefficient R was 0.93359. It indicated that the trained RBF-NN model can effectively present a positive relation between goal and output [33]. Furthermore, R values of 0.98893 and 0.99666 were obtained in the validation and test, respectively, of the trained RBF-NN model, both of which verify the effectiveness of the trained RBF-NN model for normal force. Finally, the total performance of the trained RBF-NN model for the normal force obtained an R value of 0.93575, which means the value of goodness of fit reached about 0.875. In summary, the trained RBF-NN model can fit and predict normal force very well, and the goodness of fit is greater than 87.5%.

**Figure 8:**
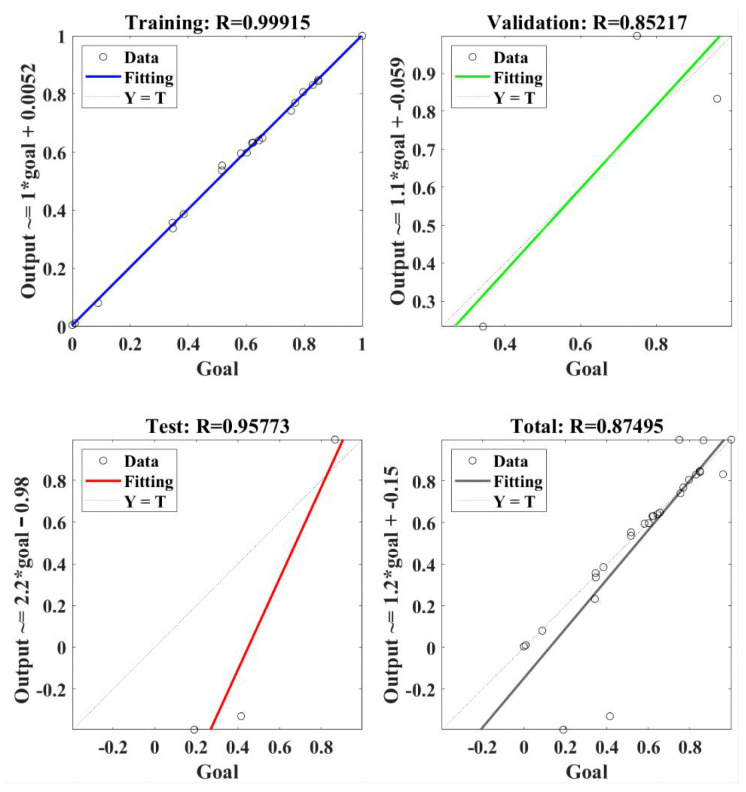
The regression of RBF-NN model for the tangential force.

The regression model of RBF-NN for the tangential force is presented in Figure 8. The RBF-NN model was trained using selected data, and the value of correlation coefficient R was 0.99915. It indicated that the trained RBF-NN model can effectively present a positive relation between goal and output. Furthermore, R values of 0.85217 and 0.95773 were obtained in the validation and test, respectively, of the trained RBF-NN model [34], both of which verify the effectiveness of the trained RBF-NN model for tangential force. Finally, the total performance of the trained RBF-NN model for the tangential force obtained an R value of 0.87495, which means the value of goodness of fit reached about 0.765. In summary, the trained RBF-NN model can fit and predict tangential force well, and the goodness of fit is about 76.5%.

**Figure 9:**
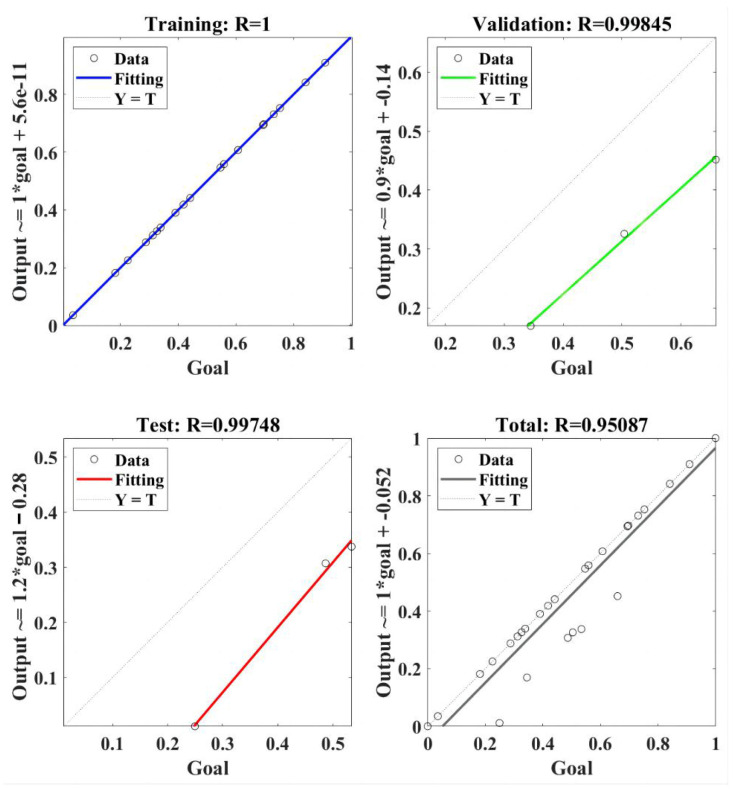
The regression of the RBF-NN model for the lateral force.

The regression model of RBF-NN for the lateral force is presented in Figure 9. The RBF-NN model was trained using selected data, and the value of correlation coefficient R was 1. It indicated that the trained RBF-NN model can effectively present a positive relation between goal and output [35]. Furthermore, R values of 0.99845 and 0.99748 were obtained in the validation and test, respectively, of the trained RBF-NN model, both of which verify the effectiveness of the trained RBF-NN model for lateral force. Finally, the total performance of the trained RBF-NN model for the lateral force obtained an R value of 0.95087, which means the value of goodness of fit reached about 0.904. In summary, the trained RBF-NN model can fit and predict lateral force very well, and the goodness of fit is about 90.4%.

**Figure 10:**
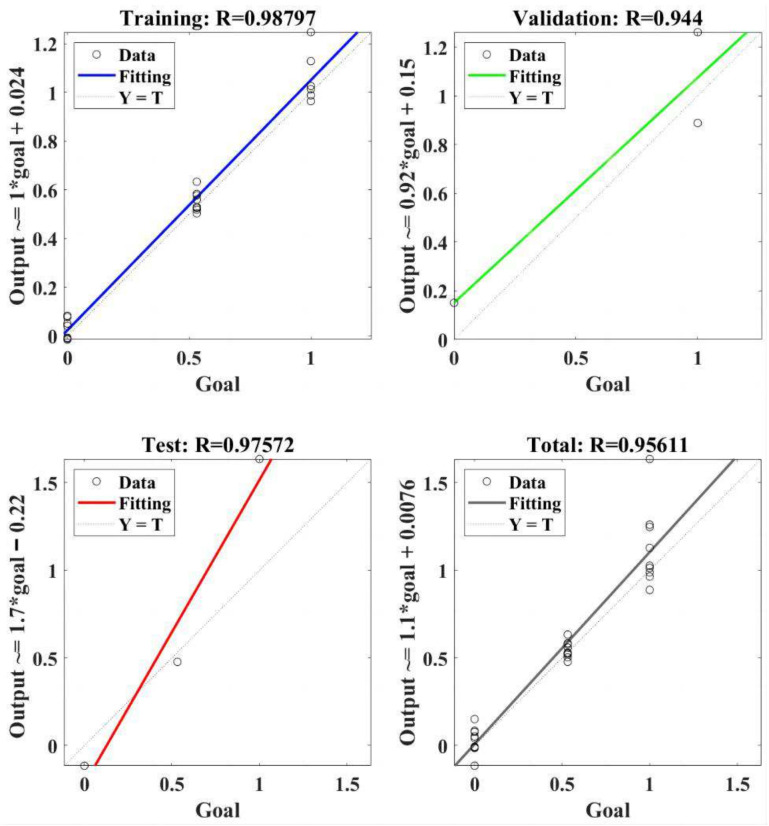
The regression of the RBF-NN model for the resultant cutting force.

The regression model of RBF-NN for the resultant cutting force is presented in Figure 10. The RBF-NN model was trained using selected data, and the value of correlation coefficient R was 0.98797. It indicated that the trained RBF-NN model can effectively present a positive relation between goal and output. Furthermore, R values of 0.944 and 0.97572 were obtained in the validation and test, respectively, of the trained RBF-NN model, both of which verify the effectiveness of the trained RBF-NN model for resultant cutting force. Finally, the total performance of the trained RBF-NN model for the resultant cutting force obtained an R value of 0.95611, which means the value of goodness of fit reached about 0.914. In summary, the trained RBF-NN model can fit and predict resultant cutting force very well, and the goodness of fit is greater than 90%.

#### 3.3.2. RBF-NN Model for the Cutting Temperature

**Figure 11:**
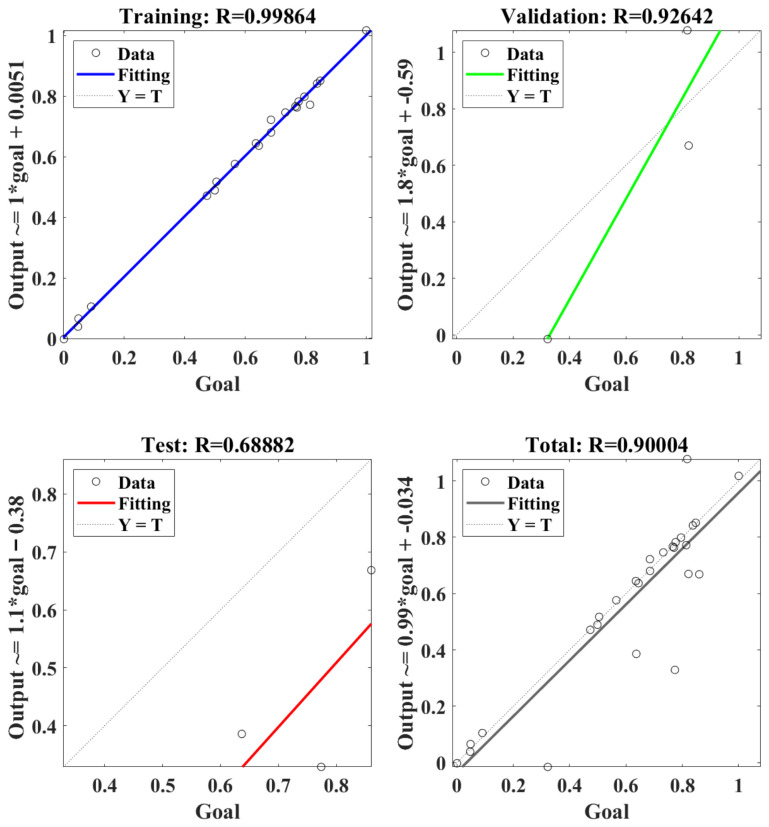
The regression of the RBF-NN model for the cutting temperature.

Figure 11 shows the regression model of RBF-NN for the cutting temperature. Firstly, the RBF-NN model was trained using selected data, and an excellent R value of 0.99864 was obtained. This means that the trained RBF-NN model can effectively show a positive relation between goal and output. Furthermore, R values of 0.92642 and 0.68882 were obtained in the validation and test, respectively, of the trained RBF-NN model, both of which verify the effectiveness of the trained RBF-NN model for cutting temperature. Finally, the total performance of the trained RBF-NN model for the cutting temperature obtained an R value of 0.90004, which means the value of goodness of fit reached about 0.81. In summary, the trained RBF-NN model can fit and predict cutting temperature well; though the goodness of fit is larger than 81%, it is lower than that of 90% resulting from cutting force.

With standardized inputs and tuned basis parameters, the RBF-NN achieved acceptable accuracy for temperature on the aggregate evaluation, consistent with the reported correlation values. The higher predictability of force relative to temperature is plausible because force integrates over volumetric material removal and is less sensitive to emissivity assumptions and field-of-view effects that can add noise to infrared measurements. Error analysis revealed larger residuals at the largest *a_e_*, suggesting that local model refinement (e.g., additional centers) could improve accuracy in high-load regimes.

The lower association for *T* compared with *F_R_* is expected for three reasons. (i) Sensing variability. Infrared thermography on WPC is sensitive to emissivity heterogeneity (wood–powder distribution, surface micro-finish), reflected apparent radiation, and ROI drift as the hotspot moves with sliding velocity; these effects inflate measurement variance relative to force acquisition. (ii) Physics and history-dependence. The interface temperature depends not only on the instantaneous [*U*, *a_e_*, *n*] but also on recent thermal history (heat accumulation, chip evacuation and airflow, local friction changes), which are not explicit inputs in the present surrogate; by contrast, forces respond more directly to instantaneous chip thickness and engagement. (iii) Data coverage and parsimony. To avoid overfitting, smaller-capacity models were selected; residuals concentrate at large *a_e_* and high *U* where both nonlinearity and heteroscedastic noise increase, limiting attainable R^2^ under a compact dataset.

Without expanding the experimental matrix, temperature prediction can be strengthened by (a) locally increasing the center density in the high-load corner (larger K only in that region), (b) adding light-weight context features readily available during testing (e.g., cumulative cutting length/pass time, ambient temperature, a force-derived wear proxy), and (c) tightening ROI control (registration to a fixed patch with high-ε coating). These are compatible with our small-sample protocol and preserve the study’s scope.

### 3.4. Comparison with Prior Reports and Implications

Most prior WPC studies focus on peripheral milling or orthogonal cutting, frequently emphasizing single-response targets (e.g., force or roughness) and relying on larger datasets. The present study centers on face milling, jointly analyzing forces and temperature under small-sample constraints (27 conditions, *N* = 3 repeats each), thereby addressing an underrepresented but industrially relevant operation for planar finishing.

The observed directions match established polymer/composite trends: forces rise with *U* and *a_e_* and decline with increasing n; temperature increases with all three factors over the tested ranges, with the increase at higher n attributed to frictional heating at greater sliding velocity. The dominance of *a_e_* over *U* in force growth agrees with engagement-area arguments reported for composite cutting, whereas the mitigating role of n on forces, but not on temperature, aligns with the softening-versus-heating dichotomy documented in polymer machining.

Compared with ANN/RBF studies that report multiple error metrics on larger datasets, the present work adopts a compact, reproducible RBF-NN protocol geared to small samples: inputs are standardized; centers and widths are defined by simple geometric rules with neighbor count *d* ∈ {3,4,5}; capacity is regulated by *K* and *λ*; and performance is communicated via R^2^ with leakage-free validation. Despite the small sample, the model attains stable association for force and acceptable association for temperature, which is consistent with the experimental variability and with prior observations that thermal signals in polymers are harder to predict than force due to emissivity and interface-friction sensitivity.

The key contribution is to systematize face-milling responses in WPC (forces + temperature) and to provide a transparent, small-sample modeling recipe that others can replicate with minimal setup. The unified presentation of effects, interactions, and trade-offs, together with explicit confidence intervals and a concise RBF-NN protocol, complements prior larger-scale studies and fills a methodological gap for scenarios where design-of-experiment budgets are constrained.

The tested ranges do not include extremely high cutting speeds or multi-pass thermal accumulation; temperature fields were measured via IR thermography with calibrated emissivity, yet local emissivity heterogeneity may remain. Future work can extend to surface integrity metrics and to targeted sampling near the high-load corner to refine temperature prediction while preserving the small-sample philosophy.

## 4. Conclusions

This study examined face milling of PVC-rich WPC with a dual focus on process responses (cutting forces and cutting temperature) and a compact, reproducible RBF-NN surrogate suitable for small datasets. Beyond confirming the monotonic trends of the factors, the combined experimental–modeling analysis clarifies why those trends arise and how they should inform process choices under practical constraints.

The results indicate a force–temperature trade-off that must be managed explicitly. Increasing *a_e_* and *U* raises forces (and temperature), whereas increasing n lowers forces but elevates temperature due to higher sliding velocity and frictional heating. For day-to-day planning, three decision regimes emerge:

(1) Force-lean regime (tool load, chatter risk): prioritize higher *n* with moderate *U* and lower *a_e_*.
(2) Temperature-lean regime (thermal limits, polymer softening): prefer lower *a_e_* and moderate *n*, with moderate-to-low *U*.
(3) Balanced regime (general finishing): operate within a *n* ≥ 8000 rpm, *a_e_* ≤ 0.2 mm, *U* ≤ 8 m/min window; tune locally based on tool wear and surface requirements.

Implications for modeling under small samples. With 27 conditions (each *N* = 3 repeats), a capacity-controlled RBF-NN (small *K*, locality via neighbor count *d* ∈ {3,4,5}, ridge regularization *λ*) achieved stable association for resultant force and acceptable association for temperature using R^2^ as a scale-independent metric.

Broader relevance and reproducibility. The factor directions and their relative influence (*a_e_* > *U* > *n* for forces) align with polymer-composite cutting mechanics, suggesting that the planning logic above will generalize to similar WPC formulations, provided emissivity calibration and ROI control are maintained.

The present ranges do not cover very high cutting speeds or multi-pass thermal accumulation; surface integrity (e.g., roughness, defects) was outside scope; the tool geometry and WPC composition were fixed. Highest-impact extensions are (i) multi-objective process optimization coupling forces/temperature with surface integrity; (ii) targeted sampling near the high-load corner to tighten thermal prediction; (iii) incorporation of informative covariates (e.g., wear state, ambient conditions) when available; and (iv) exploration of transfer learning to adjacent WPC grades and tool geometries.

## Figures and Tables

**Table 1:** Physical property of the WPC workpieces.

Workpieces	Density (g/cm^3^)	Tensile Strength (MPa)	Elasticity Strength (MPa)	Flexural Strength (MPa)	Wood Powder Particle Size (mm)
WPC	1.32	28.36	4.27 × 10^3^	4.69	0.150

**Table 2:** Property of the PCD cutter.

Cutting Tool	Thermal Conductivity (W/(m·K))	Hardness (HV)	Coefficient of Thermal Expansion
PCD cutter	560	8000	1.18 × 10^−6^

**Table 3:** Factor levels and experimental runs for face milling (*a_p_* = 5 mm).

Run	*a_e_* mm	*U* (m/min)	*n* (rpm)
1–9	0.2	6, 8, 10	6000, 8000, 10,000
10–18	0.6	6, 8, 10	6000, 8000, 10,000
19–27	1.0	6, 8, 10	6000, 8000, 10,000

## Data Availability

The original contributions presented in this study are included in the article. Further inquiries can be directed to the corresponding author.

## References

[1] Xiong X., Yue X., Wu Z. (2023). Current status and development trends of Chinese intelligent furniture industry. J. Renew. Mater..

[2] Dong W., Xiong X., Ma Y., Yue X. (2021). Woodworking tool wear condition monitoring during milling based on power signals and a particle swarm optimization-back propagation neural network. Appl. Sci..

[3] Kaymakci A., Gulec T., Hosseinihashemi S.K., Ayrilmis N. (2017). Physical, mechanical and thermal properties of wood/zeolite/plastic hybrid composites. Maderas. Cienc. Tecnol..

[4] Bourai K., Riedl B., Rodrigue D. (2013). Effect of temperature on the thermal conductivity of wood-plastic composites. Polym. Polym. Compos..

[5] Zhu Z., Buck D., Wu Z., Yu Y., Guo X. (2023). Frictional behaviour of wood-Plastic composites against cemented carbide during sliding contact. Wood Mater. Sci. Eng..

[6] Wang J., Jiang R., Wu Z., Zhu Z., Yang L., Cao P. (2023). Investigation of surface integrity up-milling magnesium oxide particle reinforced wood-based composite. Int. J. Precis. Eng. Manuf..

[7] Guo X., Wang J., Buck D., Zhu Z., Guo Y. (2021). Machinability of wood fiber/polyethylene composite during orthogonal cutting. Wood Sci. Technol..

[8] Zhu Z., Buck D., Wu Z., Wang J., Guo X., Zhu M. (2022). Built-up edge formation mechanisms in orthogonal cutting of wood-plastic composite. Wood Mater. Sci. Eng..

[9] Pimenov D.Y., Hassui A., Wojciechowski S., Mia M., Magri A., Suyama D.I., Bustillo A., Krolczyk G., Gupta M.K. (2019). Effect of the relative position of the face milling tool towards the workpiece on machined surface roughness and milling dynamics. Appl. Sci..

[10] Li R., He C., Xu W., Wang X. (2023). Modeling and optimizing the specific cutting energy of medium density fiberboard during the helical up-milling process. Wood Mater. Sci. Eng..

[11] Li R., Cao P., Zhang S., Xu W., Ekevad M., Guo X. (2017). Predicting of cutting force during gypsum fiber composite milling process using response surface methodology. Wood Fiber Sci..

[12] Li X., Zhang M., Yang L., Yue X., Xiong X. (2025). Current state and development trend of China’s customized home furnishing industry. Wood Mater. Sci. Eng..

[13] Yue X., Xiong X., Zhang M., Xu X., Yang L. (2025). Multi-objective optimization for energy-efficient hybrid flow shop scheduling problem in panel furniture intelligent manufacturing with transportation constraints. Expert Syst. Appl..

[14] Xu X., Xiong X., Yue X., Zhang M. (2023). A parametric optimized method for three-dimensional corner joints in wooden furniture. Forests.

[15] Zhang M., Xiong X., Yue X., Xu X. (2024). Status of China’s wooden-door industry and challenges lying ahead. Wood Mater. Sci. Eng..

[16] Yue X., Xiong X., Xu X., Zhang M. (2024). Big data for furniture intelligent manufacturing: Conceptual framework, technologies, applications, and challenges. Int. J. Adv. Manuf. Technol..

[17] Niu Y., Xiong X. (2022). Investigation on panel material picking technology for furniture in automated raw material warehouses. BioResources.

[18] Wu Y.-C., Feng J.-W. (2018). Development and application of artificial neural network. Wirel. Pers. Commun..

[19] Cus F., Zuperl U. (2006). Approach to optimization of cutting conditions by using artificial neural networks. J. Mater. Process. Technol..

[20] Kara F., Aslantaş K., Cicek A. (2016). Prediction of cutting temperature in orthogonal machining of AISI 316L using artificial neural network. Appl. Soft Comput..

[21] Sonar D., Dixit U., Ojha D. (2006). The application of a radial basis function neural network for predicting the surface roughness in a turning process. Int. J. Adv. Manuf. Technol..

[22] Szwajka K., Zielińska-Szwajka J., Trzepieciński T. (2023). The use of a radial basis function neural network and fuzzy modelling in the assessment of surface roughness in the MDF milling process. Materials.

[23] Garg S., Pal S.K., Chakraborty D. (2007). Evaluation of the performance of backpropagation and radial basis function neural networks in predicting the drill flank wear. Neural Comput. Appl..

[24] Garg S., Patra K., Khetrapal V., Pal S.K., Chakraborty D. (2010). Genetically evolved radial basis function network based prediction of drill flank wear. Eng. Appl. Artif. Intell..

[25] Srinivasa P., Nagabhushana T., Ramakrishna Rao P. (2002). Flank wear estimation in face milling based on radial basis function neural networks. Int. J. Adv. Manuf. Technol..

[26] Nariman-zadeh N., Darvizeh A. (2003). Hybrid genetic learning of radial basis function networks (RBFNs) for the modeling of the explosive cutting process of plates. Sci. Iran..

[27] Li B.D. (2011). Cutting parameters optimization based on radial basis function neural network and particle swarm optimization. Adv. Mater. Res..

[28] Tsao C. (2008). Prediction of thrust force of step drill in drilling composite material by Taguchi method and radial basis function network. Int. J. Adv. Manuf. Technol..

[29] Shan K., Zhang Y., Lan Y., Jiang K., Xiao G., Li B. (2023). Surface Roughness Prediction of Titanium Alloy during Abrasive Belt Grinding Based on an Improved Radial Basis Function (RBF) Neural Network. Materials.

[30] Nguyen T.-T., Dang X.-B. (2025). Radial basis function network-based optimization of the hard self-propelled rotary turning titanium. Neural Comput. Appl..

[31] Cuevas E., Ascencio-Piña C.R., Pérez M., Morales-Castañeda B. (2024). Considering radial basis function neural network for effective solution generation in metaheuristic algorithms. Sci. Rep..

[32] Tang X.P., Wang D.Y. (2014). Predication of rock cutting force of conical pick based on RBF neural network. Appl. Mech. Mater..

[33] Wang J., Wu Z., Zhang F., Song C., Hu W., Zhu Z., Guo X., Cao P. (2024). Research on the end-milling surface quality of paulownia based on response surface model in terms of force and chip morphology. Forests.

[34] Wang J., Buck D., Tang Q., Guan J., Zhou X., Wu Z., Cao P., Guo X., Zhu Z. (2023). Machining properties of stone-plastic composite based on an empirically validated finite element method. Adv. Eng. Mater..

[35] Jin D., Wei K. (2021). Machinability of Scots pine during peripheral milling with helical cutters. BioResources.

